# Multiple association analysis of loci and candidate genes that regulate body size at three growth stages in Simmental beef cattle

**DOI:** 10.1186/s12863-020-0837-6

**Published:** 2020-03-14

**Authors:** Bingxing An, Lei Xu, Jiangwei Xia, Xiaoqiao Wang, Jian Miao, Tianpeng Chang, Meihua Song, Junqing Ni, Lingyang Xu, Lupei Zhang, Junya Li, Huijiang Gao

**Affiliations:** 1 Institute of Animal Science, Chinese Academy of Agricultural Science, Beijing, 100193 China; 2 Institute of Basic Medical Sciences, Westlake Institute for Advanced Study, Hangzhou, 310000 China; 3 Zhuang Yuan Veterinary Station of Qixia city, Yantai, 265300 China; 4 Heibei Livestock Breeding Workstation, Shijiazhuang, 050061 China

**Keywords:** Simmental beef cattle, Genome-wide association studies, Body size, Candidate genes, Bovine HD 770 K SNP

## Abstract

**Background:**

Body size traits as one of the main breeding selection criteria was widely used to monitor cattle growth and to evaluate the selection response. In this study, body size was defined as body height (BH), body length (BL), hip height (HH), heart size (HS), abdominal size (AS), and cannon bone size (CS). We performed genome-wide association studies (GWAS) of these traits over the course of three growth stages (6, 12 and 18 months after birth) using three statistical models, single-trait GWAS, multi-trait GWAS and LONG-GWAS. The Illumina Bovine HD 770 K BeadChip was used to identify genomic single nucleotide polymorphisms (SNPs) in 1217 individuals.

**Results:**

In total, 19, 29, and 10 significant SNPs were identified by the three models, respectively. Among these, 21 genes were promising candidate genes, including *SOX2, SNRPD1, RASGEF1B, EFNA5, PTBP1, SNX9, SV2C, PKDCC, SYNDIG1, AKR1E2,* and *PRIM2* identified by single-trait analysis; *SLC37A1, LAP3, PCDH7, MANEA,* and *LHCGR* identified by multi-trait analysis; and *P2RY1, MPZL1, LINGO2, CMIP,* and *WSCD1* identified by LONG-GWAS.

**Conclusions:**

Multiple association analysis was performed for six growth traits at each growth stage. These findings offer valuable insights for the further investigation of potential genetic mechanism of growth traits in Simmental beef cattle.

## Background

In China, the production of beef cattle is a very important agribusiness, and the Simmental breed accounts for more than 70% of beef-producing herds. Beef producers use body size to monitor the growth of each animal throughout the fattening period [1, 2], as this trait is an indicator of cattle [3] and longevity [4]. Monitoring the development of each animal can help to increase profits by enhancing the efficiency of feed and management [5–7]. Besides in human, additive genetic effect explains 81% of the variation in height [8], and the heritability for both hip height (HH) and height size (HS) is 0.33–0.4 in cattle [9]. Bouwman et al. reported that the lead variants in significant regions explained at most 13.8% of the phenotypic variance in their meta-analysis of 58,265 cattle from 17 populations [10]. In addition, daily body linear measurements, specifically body height (BH) and HH, two highly reliable and accurate indicators for body weight, are easier to obtained than body weight [11]. Furthermore, good depth of HS in cattle is a sign of good feet and leg conformation [12], and dairy cows with higher HH will subsequently have better milk performance [13]. However, there is little information on the molecular mechanisms of body size traits in Chinese Simmental beef cattle.

Genome-wide association studies (GWAS) are robust statistical tools are that broadly identify candidate genes with significant SNPs involved in production traits [14–16], growth traits [17, 18], carcass quality traits and fertility traits [19, 20]. In beef cattle, various SNPs, genes, and haplotype blocks have been found to associate with growth, however the current GWAS-based studies focus mainly on only one growth parameter [21], such as the weaning size [22], yearling weight or stature upon slaughter [23]. Furthermore, loci controlling growth traits may be variable in different growth stages, and some loci may control traits throughout the lifetime of the animal [24]. Therefore, it is more reasonable to perform GWAS on growth traits on each stage separately. Multi-trait methods have been developed to increase statistical power and to identify pleiotropic loci in GWAS [25]. The longitudinal GWAS consider all time points when assessing whether significant SNPs associate with trait development over time [26], and this method is powerful for identifying these time-dependent and consistent loci [27]. We performed multiple trait GWAS (multi-trait GWAS) and longitudinal-GWAS (LONG-GWAS) based on single-trait analysis. Multi-trait GWAS and LONG-GWAS were not replacements for the single-trait GWAS; instead, they complemented single-trait GWAS. Thus, understanding the genetic mechanisms involved in inter-individual variations in body size may provide new insights that can help to manipulate cattle growth and production.

In this study, six body size traits were routine measured from the time cattle entering farm to slaughter, which provides valuable resources to study the complete growing period. The aim of our study was to comprehensively analyze of candidate genes and QTL regions associated with growth traits by conducting three GWAS approaches in Simmental beef cattle. Our findings offer valuable insights for the further investigation of the potential mechanism of growth traits in Simmental beef cattle.

## Results

### Population stratification assessment

**Fig. 1:**
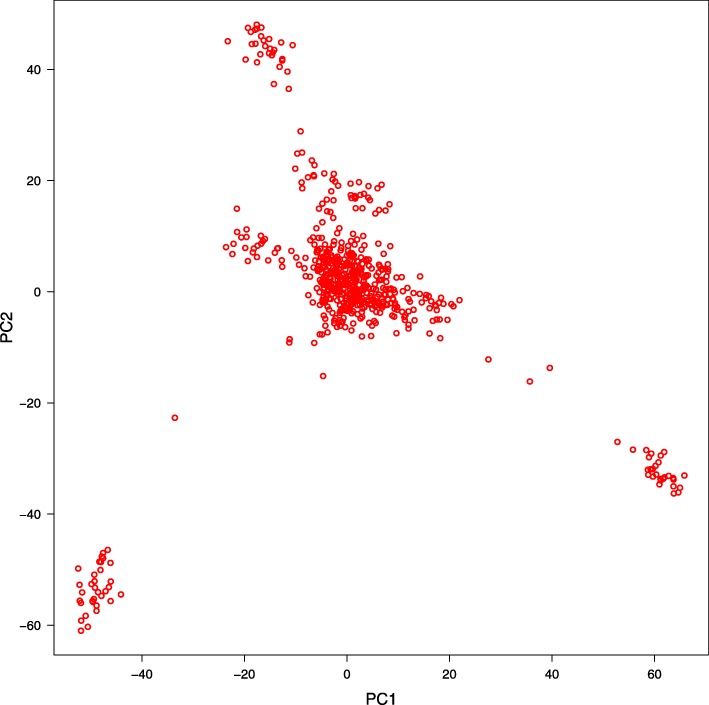
Principal components (PC) plot drawn from the second principal component (PC2) against the first principal component (PC1)

**Fig. 2:**
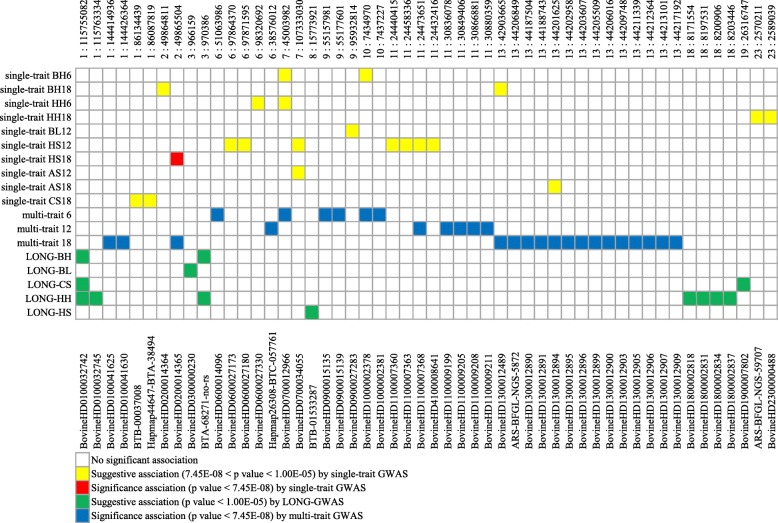
Summary of six body size traits associations across genomic regions (SNPs) with three association strategies. Each row represents a trait, and each column, a genomic region containing SNPs that are genome-wide suggestively or significantly associated with a trait. Only traits with at least one associated SNP and SNPs associated with at least one trait are shown

Figure 1 shows that the population stratification of the Simmental population based on the PCA was divided into five separate clusters, demonstrating an obvious stratification in the reference population. The population stratification caused by different genetic influences and breeding conditions, as potential confounders, was corrected by significance testing. We summarized the genome-wide significant and suggestive SNP regions for these traits in Fig. 2. The Manhattan plots and quantile-quantile (Q-Q) plots are shown in Figure S1 and Figure S2, while Q-Q plots suggested that there was no inflation or systematic bias in this research. Most points were near diagonal line because the GWAS model sufficiently considered the population structure and only a few SNPs were associated with the target traits. Meanwhile, the genomic inflation factors (λ) at each trait ranged from 1.03 to 1.10, indicating consistent consequence with PCA.

### Summary of significant loci identified by three approaches

Briefly, we found 45, 66 and 19 SNPs significantly were associated with six body size traits by single-trait GWAS, multi-trait GWAS and LONG-GWAS, respectively. There were no significant loci for single-trait BH6 (single-trait GWAS for BH at 6 months after birth, and so forth), single-trait AS6, single-trait CS6, and LONG-AS. In addition, ten SNPs were associated with at least one of the six traits and eight SNPs were strongly associated with these traits in at least one of the three models. While according to their biological functions, 21 suggestive genes were selected as candidate genes and some details of them, including their positions in the genome, the nearest reported genes, the minor allele frequencies (MAF) and the *p* values are listed in Table 1.

**Table 1:** List of suggestive candidate genes associated with six body size traits in Simmental beef cattle

Genes	Related SNPs	BTA	Position (bp)	MAF	Distance (bp)	*p* value	Associated traits
P2RY1	BovineHD0100032742	1	115,755,082	0.40	79,694	9.51E-06	LONG-BH
	BovineHD0100032742	1	115,755,082	0.40	79,694	1.54E-06	LONG-HH
	BovineHD0100032742	1	115,755,082	0.40	79,694	9.75E-06	LONG-CS
	BovineHD0100032745	1	115,763,334	0.40	71,442	1.99E-06	LONG-HH
SLC37A1	BovineHD0100041625	1	144,414,936	0.24	within	7.32E-08	multi-trait18
	BovineHD0100041630	1	144,426,364	0.24	within	7.32E-08	multi-trait18
SOX2	BTB-00037008	1	86,134,439	0.21	200,052	8.34E-06	single-trait CS18
	Hapmap44647-BTA-38494	1	86,087,819	0.19	153,432	3.58E-06	single-trait CS18
SNRPD1	BovineHD0200014364	2	49,864,811	0.20	128,412	9.22E-06	single-trait HS18
	BovineHD0200014365	2	49,865,504	0.25	129,105	2.11E-08	single-trait HS18
	BovineHD0200014365	2	49,865,504	0.24	129,105	4.06E-08	multi-trait18
MPZL1	BovineHD0300000230	3	966,159	0.38	within	7.60E-06	LONG-BL
	BTA-68271-no-rs	3	970,386	0.42	within	8.93E-07	LONG-BH
	BTA-68271-no-rs	3	970,386	0.42	within	6.64E-06	LONG-HH
PCDH7	BovineHD0600014096	6	51,063,986	0.00	472,877	4.90E-08	multi-trait6
RASGEF1B	BovineHD0600027173	6	97,864,370	0.15	125,438	8.09E-06	single-trait HS12
	BovineHD0600027180	6	97,871,595	0.15	118,213	4.49E-06	single-trait HS12
	BovineHD0600027330	6	98,320,692	0.10	306,357	9.24E-06	single-trait HH6
LAP3	Hapmap26308-BTC-057761	6	38,576,012	0.39	within	1.94E-08	multi-trait12
PTBP1	BovineHD0700012966	7	45,003,982	0.02	12,339	3.90E-07	single-trait HH6
	BovineHD0700012966	7	45,003,982	0.02	12,339	7.42E-06	single-trait BH6
	BovineHD0700012966	7	45,003,982	0.02	12,339	8.23E-13	multi-trait6
EFNA5	BovineHD0700034055	7	107,333,030	0.00	1,716,560	1.84E-06	single-trait HS12
	BovineHD0700034055	7	107,333,030	0.00	1,716,560	7.68E-06	single-trait AS12
LINGO2	BTB-01533287	8	15,773,921	0.31	495,444	7.98E-06	LONH-HS
MANEA	BovineHD0900015135	9	55,157,981	0.01	within	5.01E-08	multi-trait6
	BovineHD0900015139	9	55,177,601	0.01	within	5.01E-08	multi-trait6
SNX9	BovineHD0900027283	9	95,932,814	0.23	within	5.18E-06	single-trait BL12
SV2C	BovineHD1000002378	10	7,434,970	0.35	within	4.16E-07	single-trait BH6
	BovineHD1000002378	10	7,434,970	0.35	within	2.99E-09	multi-trait6
	BovineHD1000002381	10	7,437,227	0.14	within	5.96E-08	multi-trait6
PKDCC	BovineHD1100007360	11	24,440,415	0.30	89,224	2.65E-06	single-trait HS12
	BovineHD1100007363	11	24,458,336	0.30	71,303	4.35E-06	single-trait HS12
	BovineHD1100007368	11	24,473,651	0.49	55,988	3.84E-06	single-trait HS12
	BovineHD1100007368	11	24,473,651	0.50	55,988	2.88E-08	multi-trait12
	BovineHD4100008641	11	24,432,416	0.29	97,223	4.62E-06	single-trait HS12
LHCGR	BovineHD1100009199	11	30,836,078	0.42	within	2.57E-08	multi-trait12
	BovineHD1100009205	11	30,849,406	0.43	within	4.07E-08	multi-trait12
	BovineHD1100009208	11	30,866,881	0.43	within	4.62E-08	multi-trait12
	BovineHD1100009211	11	30,880,359	0.43	within	4.07E-08	multi-trait12
SYNDIG1	BovineHD1300012489	13	42,903,665	0.43	10,485	1.06E-06	single-trait BH18
	BovineHD1300012489	13	42,903,665	0.43	10,485	6.31E-08	multi-trait18
AKR1E2	ARS-BFGL-NGS-5872	13	44,206,849	0.49	within	4.17E-09	multi-trait18
	BovineHD1300012890	13	44,187,504	0.50	16,132	3.20E-09	multi-trait18
	BovineHD1300012891	13	44,188,743	0.50	14,893	4.89E-09	multi-trait18
	BovineHD1300012894	13	44,201,625	0.46	2011	8.08E-06	single-trait AS18
	BovineHD1300012894	13	44,201,625	0.47	2011	3.84E-10	multi-trait18
	BovineHD1300012895	13	44,202,958	0.50	678	4.65E-08	multi-trait18
	BovineHD1300012896	13	44,203,607	0.50	29	6.37E-09	multi-trait18
	BovineHD1300012899	13	44,205,509	0.50	within	2.75E-09	multi-trait18
	BovineHD1300012900	13	44,206,016	0.49	within	4.18E-09	multi-trait18
	BovineHD1300012903	13	44,209,748	0.50	within	2.75E-09	multi-trait18
	BovineHD1300012905	13	44,211,339	0.49	within	2.62E-08	multi-trait18
	BovineHD1300012906	13	44,212,364	0.49	within	3.11E-09	multi-trait18
	BovineHD1300012907	13	44,213,101	0.48	within	5.66E-09	multi-trait18
	BovineHD1300012909	13	44,217,192	0.48	2915	2.63E-08	multi-trait18
CMIP	BovineHD1800002818	18	8,171,554	0.48	39,261	5.92E-06	LONG-HH
	BovineHD1800002831	18	8,197,531	0.43	13,284	6.66E-06	LONG-HH
	BovineHD1800002834	18	8,200,906	0.43	9909	5.72E-06	LONG-HH
	BovineHD1800002837	18	8,203,446	0.43	7369	4.87E-06	LONG-HH
WSCD1	BovineHD1900007802	19	26,316,747	0.37	31,164	5.47E-06	LONG-CS
PRIM2	ARS-BFGL-NGS-59707	23	2,570,211	0.36	within	6.65E-06	single-trait HH18
	BovineHD2300000488	23	2,589,039	0.36	within	8.16E-06	single-trait HH18

Name of trait: *BH* body height; *BL* body length; *HH* hip height; *HS* heart size; *AS* abdominal size; *CS* cannon bone size

Name of SNPs: Single nucleotide polymorphism name in the Bovine HD panel

*BTA Bos Taurus* autosome

*MAF* minor allele frequency

Position: Position (bp) on UMD3.1

Distance: distance between SNP and the nearest gene

*P* value: *p*-values calculated from the mixed linear model analysis

### SNPs identified by single-trait GWAS

A total of 45 SNPs achieved genome-wide significance associated with at least one of the six traits, with the *p*-value ranging from 9.99 × 10^− 6^ (BovineHD0700018941 for BL18) to 2.11 × 10^− 8^ (BovineHD0200014365 for HS18), and the MAF ranging from 0.003 (BovineHD0700034055) to 0.497 (BovineHD2600012755). Among these, two SNPs near *SOX2* (SRY-Box 2) on BTA1 were also identified in the liver and stomach [28]. On BTA2, two loci in the 0.69 Kb region were significantly associated with single-trait HS18 and one of them (BovineHD0200014365) was also associated with multi-trait18. On BTA6, three SNPs in the 0.46 Mb region were located near *RASGEF1B* (RasGEF Domain Family Member 1B). On BTA7, one SNP (BovineHD0700034055) was associated with single-trait HS12 and single-trait AS12, namely *EFNA5* and another SNP (BovineHD0700012966) was associated with single-trait HH6 and multi-trait6, namely *PTBP1*. On BTA10, one SNP (BovineHD1000002378) was associated with single-trait BH6 and multi-trait6, namely *SV2C*. While on BTA11, four loci in 0.04 Mb region were associated with the single-trait HS12 and one of them (BovineHD1100007368) was also associated with multi-trait12, all of which were near *PKDCC*. On BTA13, SNP BovineHD1300012489 and BovineHD1300012894 were associated with single-trait BH18 and single-trait AS18, respectively. These two SNPs also strongly associated with multi-trait18. On BTA23, two SNPs were found within *PRIM2*.

### SNPs identified by multi-trait GWAS

Multi-trait GWAS identified 66 SNPs within or near 36 genes that were distributed on 21 chromosomes, including 8 loci that were also identified by single-trait GWAS, which indicated that these loci suggestively regulate the development of the body growth (Table 1). Among these, two promising loci within the 11.4 Kb region were detected, namely *SLC37A1*. On BTA6, two suggestive loci were detected, one near *LAP3* that associated with multi-trait12 and another near *PCDH7*. Two genome-wide loci were identified within the 0.02 Mb region of *MANEA* on BTA9. Furthermore, four promising loci were detected within in 0.04 Mb region of *LHCGR* on BTA11, and 13 loci were identified within the 0.03 Mb region of *AKR1E2* on BTA13, which were also detected by single-trait analysis.

### SNPs identified by LONG-GWAS

Nineteen loci were identified by LONG-GWAS, including three significant loci on 12 chromosomes (Table 1). Among them, two suggestive loci in the 8.3Kb region near *P2RY1* were detected, whereas the other (BovineHD0100032742) was also associated with LONG-CS, LONG-BH and LONG-HH. Another two loci in the 4.2 Kb region near *MPZL1* were identified, and the latter SNP (BTA-68271-no-rs) was also associated with LONG-HH and LONG-BH. Furthermore, one suggestive SNP near *LINGO2* on BTA8 was associated with LONG-HS. In addition, four promising loci in the 0.03 Kb region were associated with LONG-HH, namely *CMIP*, a key gene in the T-cell signaling pathway. On BTA19, a suggestive locus near *WSCD1* was associated with LONG-CS. No loci were associated with AS in our research.

## Discussion

We performed single-trait GWAS, multi-trait GWAS, and LONG-GWAS for six body size traits on three growth stages in Simmental beef cattle. However, the three methods yielded different results with few shared loci. The reason for this discrepancy was likely due to the restricted dataset in single-trait GWAS and LONG-GWAS analyses. One universal phenomenon that cannot be ignored is that growth traits are controlled by multiple genes [29], and each method had its specific advantages in the identification of distinct loci. For example, single-trait GWAS is robust in detecting trait-specific QTLs and multi-trait GWAS is efficient for mapping pleiotropic QTLs [30], whereas LONG-GWAS can improve the detection power for time-dependent and consistent loci [31]. Thus, combining these three GWAS methods was expected to markedly improve the analysis the genetic mechanism of the body traits of beef cattle. In addition, since many complex traits have a similar architecture across diverse species [7], which prompted us to compare some of our significant genes with the previous reports that investigate the same genes and their involvements in growth. As a result, 21 suggestive genes were considered as candidate genes that were involved in the growth of cattle, swine, mice, and humans.

### Candidate genes identified by single-trait GWAS

On BTA1, two SNPs were near *SOX2* (SRY-Box 2), which encodes a transcription factor involved in the regulation of embryonic development [32, 33]. The paralog of this gene is *SOX17*, which positively affects the growth traits of cattle, and the conserved regions of this gene in human genome is closely related to body development [34]. On BTA2, two SNPs were near *SNRPD1* (small nuclear ribonucleoprotein D1 polypeptide), which is a member of the ghrelin receptor family, and the encoded protein is involved in zinc-dependent signaling in epithelial tissue [35]. On BTA6, variations near *RASGEF1B* (RasGEF domain family member 1B) were associated with body height [36]. In addition, body height was positively correlated with calcium absorption, which is an important determinant of calcium balance [37]. On BTA7, a SNP near *EFNA5* (ephrin A5) was associated with two traits (HS and AS) at the same stage. It was also identified as a candidate gene for growth traits in broiler chicken [38]. Another SNP near *PTBP1* (polypyrimidine tract binding protein 1) was found to show genome-wide association with growth traits at 6 months by both single-trait and multi-trait GWAS. Its expression level determined the release of insulin, thereby affecting development [39]. On BTA9, a SNP (BovineHD0900027283) located in *SNX9* (sorting nexin 9), as olfactory receptor, was associated with growth traits in Yorkshire pigs [40]. The SNP near *SV2C* (synaptic vesicle glycoprotein 2C) was associated with BH by both single-trait and multi-trait GWAS. This gene was reported to modulate dopamine release in neural and endocrine cells [41]. On BTA11, *PKDCC* (protein kinase domain containing, cytoplasmic) was associated with HS in both single-trait and multi-trait analyses. This gene was involved in the maintenance of bone density in humans [42]. On BTA13, *SYNDIG1* (synapse differentiation inducing 1) has been reported as a factor influencing the final weight and backfat thickness of Landrace pigs [43], whereas the *AKR1E2* (aldo-keto reductase family 1 member E2) variant was associated with body length and girth in cattle [44]. On BTA23, the *PRIM2* (DNA primase subunit 2) was associated with body weight and trait changes in pigs [45, 46].

### Single-trait GWAS versus multi-trait GWAS

Multiple-trait analysis of linkage experiments has been reported to significantly enhance the power to detect common SNPs across traits [47, 48]. Therefore, we used multi-trait analysis to complement single-trait GWAS rather than to replace it. In the single-trait GWAS, the minimum *p* values for the three stages were 3.90E-07, 9.92E-07 and 2.11E-08 respectively. These three values decreased to 8.23E-13, 1.73E-09 and 3.84E-10 in the multi-trait GWAS, respectively. We also identified several critical loci as follows. On BTA1, the *SLC37A1* (solute carrier family 37, member A1) gene, which encodes a glucose-6-phosphate transporter that is involved in the homeostasis of blood glucose [48], was found to be the best candidate gene for modifying milk production traits [49]. On BTA6, *LAP3* (leucine aminopeptidase 3) was reported to play critical roles in the regulation of hormone levels and protein maturation. Another study demonstrated that putative regulatory elements in the *PCDH7* (protocadherin 7) gene may have roles in residual feed intake in Nelore cattle [50]. In addition, *MANEA* (mannosidase endo-alpha), which has roles in proteolysis, was associated with the birth weight of Canchim beef cattle [17]. On BTA11, a mutation in the *LHCGR* (luteinizing hormone/choriogonadotropin receptor) gene was as the cause of empty follicle syndrome [51].

### Single-trait GWAS versus LONG-GWAS

We used LONG-GWAS, which involved multiple phenotype measurements for each individual [24]. One disadvantage of this method was that incorporating all data may have overwhelmed significant signal, that is, if QTL effects varied during the different stages [52]. In this study, these time-specific expressed QTLs identified by the single-trait and multi-trait GWAS were not detected by LONG-GWAS. However LONG-GWAS also detected some significant functional loci as follows. On BTA1, *P2RY1* (purinergic receptor P2Y1), a candidate gene that affects the serum Ca^2+^, encoded for a member of the family of G protein-coupled receptor family that works as receptor for extracellular ATP and ADP [53]. On BTA3, the *MPZL1* (myelin protein zero like 1) gene could significantly enhance the migratory and metastatic potential of hepatocellular carcinoma cells by phosphorylating and activating the pro-metastatic protein [54]. Besides on BTA8, *LINGO2* (leucine rich repeat and Ig domain containing 2), which is expressed in the central nervous system of mouse embryos, has been reported to associate with the body mass in a cohort of elderly Swedes [55]. On BTA18, *CMIP* (C-Maf inducing protein), a candidate gene for reading-related traits, was also associated with plasma lipoprotein levels [56]. Moreover, *WSCD1* (WSC domain containing 1), which encodes a protein with sulfotransferase activity that participates in the metabolism of glucose, was a candidate gene for feed efficiency and feeding behaviors in the White Duroc × Erhualian F2 population [57].

## Conclusions

In conclusion, a total of 58 SNPs corresponding to 21 genes were found to be associated with six body size traits at 6, 12 and 18 months. Future studies characterizing the functions of these candidate genes may uncover the genetic architecture underlying the body size traits in Simmental beef cattle.

## Methods

### Resource population and phenotypes collection

Simmental beef cattle (born between 2008 and 2015) were established in Ulgai, Xilingole League, Inner Mongolia of China. Six body size traits at three growth stages (6, 12, and 18 months after birth) were measured simultaneously for each individual. The details of trait measurement are as follows: BH, also named wither height, the height from wither to ground; BL, the length from the front edge of the scapula to the back edge of the ischial tuberosity; HH, height from hip to ground; HS, also named heart girth, the chest girth in the back edge of scapula; AS, the girth of the thickest part of the abdomen; CS, the girth of the thinnest cannon bone. In addition, only cattle (133 individuals, Table S1) whose phenotype records overlapped during three growth stages were used for LONG-GWAS analysis. The blood sample specimens were collected during the regular quarantine inspection of the farms was conducted. After this study, all individuals were slaughtered in strict compliance with the Institutional Meat Purchase Specifications for fresh beef. All procedures were conducted in strict compliance with the guidelines established by the Ministry of Agriculture of China. Some descriptive statistics and heritability estimates of six traits at three growth stages are presented in Table 2.

**Table 2:** Descriptive statistics of body size traits at 6, 12, and 18 months after birth

Month	Trait (cm)	N^a^	Mean	Min.	Max.	SD	*h*^2^ (SE)
6	BH	218	100.44	80	127	9.803	0.49 ± 0.04
	HH	121	105.01	85	136	10.42	0.51 ± 0.06
	BL	214	105.31	72	138	10.65	0.52 ± 0.05
	HS	213	125.44	89	170	15.61	0.51 ± 0.06
	AS	211	140.87	97	188	17.54	0.51 ± 0.07
	CS	119	16.377	12	21.5	2.323	0.62 ± 0.04
12	BH	457	116.69	97	135	7.274	0.29 ± 0.08
	HH	453	123.69	105	142	7.415	0.27 ± 0.08
	BL	453	130.18	104	157	9.541	0.53 ± 0.07
	HS	454	168.24	129	202	13.09	0.33 ± 0.05
	AS	454	198.53	155	238	15.24	0.30 ± 0.07
	CS	436	18.069	15	21.5	1.221	0.29 ± 0.06
18	BH	516	126.46	105	139	4.577	0.28 ± 0.06
	HH	267	132.61	109	147	5.453	0.41 ± 0.04
	BL	514	144.19	123	169	7.843	0.28 ± 0.07
	HS	513	188.11	160	214	8.133	0.30 ± 0.08
	AS	512	219.01	193	244	8.909	0.14 ± 0.06
	CS	381	20.155	17	23	1.506	0.54 ± 0.07

*h*^2^ heritability, *SE* standard error, *BH* body height, *BL* body length, *HH* hip height, *HS* heart size, *AS* abdominal size, *CS* cannon bone size

^a^Number of animal with phenotypes

### Genotyping and quality control

Genomic DNA was isolated from blood samples using the TIANamp Blood DNA Kit (Tiangen Biotech Co.Ltd.,Beijing, China). DNA quality was acceptable when the A260/A280 radio was between 1.8 and 2.0. Genotyping was performed with the Illumina BovineHD Beadchip (Illumina Inc., San Diego, CA, USA) and the PLINK v1.07 Software was used for quality control [58]. In this study, animals with a call rate (< 0.9) were discarded. SNPs were deleted the following standards, including minor allele frequency (< 0.01), SNP call rate (< 0.05) and Hardy-Weinberg equilibrium values (*p* < 1 × 10^− 6^). Finally, 671,192 SNPs on 29 autosomal chromosomes with an average distance of 3 kb were generated for the analysis.

### Single-trait GWAS

For six body size traits in three growth stage, we performed single-trait GWAS, respectively. The compressed mixed linear model (CMLM) was called because it reduced computing time by clustering individuals into groups, increased the power in QTN detection by eliminating the need to re-compute variance components, and enhanced the effectiveness in correcting the inflation from the polygenic background and controlling the bias of population stratification [59, 60]. Briefly, a principal components analysis (PCA) was performed and a kinship matrix was calculated using the Genome Association and Prediction Integrated Tool (GAPIT) package in R v3.4.2 [61]. To revise the effects of population structure, the **Q** matrix was reflected by the PCA. To replace the incomplete pedigrees, the **K** matrix was calculated by the VanRaden algorithm [62]. This model is as follows:

$$ \mathrm{y}=\mathrm{W}\upupsilon +\mathrm{X}\ \upbeta +\mathrm{Z}\ \mathrm{u}+\mathrm{e} $$

where ***y*** is a vector of the observed phenotypes; ***W*** was a vector of SNP genotype indicators, which was coded as 0, 1 and 2 corresponding to the three genotypes AA, AB, and BB with B being the minor allele. *υ* was the effect of marker, which is treated as a fixed effect; Variable **X** is an incidence matrix for non-genetic fixed effects, and ***β*** is a non-genetic vector of fixed effects including month ages (time of birth to measurement), enter weight (weight of just entering the farm), fattening days (time of entering farm to measurement) and principal component effects (the top three eigenvectors of the **Q** matrix). Variable **Z** is an incidence matrix for a vector of polygenic effects, and parameter ***u*** is a vector for residual polygenic effects with an assumed *N (0, Kσ*^*2*^*)* distribution, where *σ*^*2*^ is the polygenic variance and ***K*** is a marker inferred kinship matrix. While ***е*** is a vector for random residual errors with a putative *N (0, I*${\sigma}_e^2$*)* distribution, where ${\sigma}_e^2$ is the residual variance. The heritability (*h*^2^) is defined as: *h*^2^ = $\frac{\sigma^2}{\sigma^2+{\sigma}_e^2}$. The CMLM analysis was performed with GAPIT Software package (http://www.maizegenetics.net/gapit). Quantile–quantile (Q–Q) plots were generated to visualize the goodness of fitting for the GWAS model accounted by the population structure and familial relatedness. The negative logarithm of the *p* value from the model was calculated against the expected value based on the null hypothesis. The threshold p value after Bonferroni correction was 0.05/*N* = 7.45 × 10^− 8^, where N is the number of SNPs. In light of the fact that the Bonferroni correction results were too stringent with low statistical power [63]. Hence, we adopted the false discovery rate (FDR) to determine the threshold values for Single-GWAS, Multi-trait GWAS and LONG-GWAS. The FDR was set as 0.01, and the threshold p value was calculated as follows:

$$ P=\mathrm{FDR}\times n/m $$

where *n* is the number of *P* < 0.01 in the results, and *m* is the total number of SNPs [64].

### Multi-trait GWAS

For the three growth stages, the multi-trait GWAS were conducted to detect pleiotropic SNPs and the model was a Chi square statistic, which approximately followed a Chi square distribution with the number of traits tested as the number of degrees of freedom. It was calculated for each SNP using the following formula [65]:

$$ {t}_i=\frac{\left|\hat{v_i}\right|}{\sqrt{V\left(\hat{v_i}\right)}} $$

$$ \chi 2 multi- trait={t}_i^{\prime }{\mathrm{V}}^{-1}{t}_{i.} $$

where $\hat{v_i}$ is the estimate of ***v*** and the corresponding variance $V\left(\hat{v_i}\right)$ can be obtained by the compressed mixed linear model (CMLM); While *t*_*i*_ is the 6 × 1 vector of the signed t-values of the *i*th SNP from the above-mentioned single-trait GWAS for the six traits. Matrix ${t}_i^{\prime }$ is the transpose of the vector *t*_*i*_, and *V*^−1^ is the inverse of the 6 × 6 correlation matrix between traits, which was calculated by the estimated effects of the qualified SNPs (signed *t* values).

### Long-GWAS

For these individuals that completely obtained body size information at three growth stages, we conducted a longitudinal GWAS by LONG-GWAS [24]. This model was similar to CMLM, except that the phenotypic variance was partitioned to SNPs, fixed factors (the above-mentioned *β* vector), polygenic effects, time stage effects and residual variance. Moreover, numerous studies have reported that the longitudinal design could facilitate the identification of time-dependent and consistent loci, which increased the statistical power due to their effectiveness in incorporating the correlation structure of multiple measurements and alleviating the multiple testing burden [26, 27, 66]. The code data implementing this method may be found at http://genetics.cs.ucla.edu/longGWAS/. This model is as follows:

$$ {\mathrm{y}}^{\ast }=\mathrm{Wv}+\mathrm{Zu}+\mathrm{y}+\mathrm{e} $$

In this formula, ***y***^***∗***^ is the adjusted phenotype (these fixed effects mentioned in CMLM were adjusted in order to account for additional confounding). The incidence matrix ***W***, ***Z***, the vector ***v***, ***u*** and ***e*** are consistent with CMLM mentioned above. Differently, the parameter ***γ*** is a vector for time stage effects with a putative *N* (0, ${\upsigma}_v^2\boldsymbol{D}$) distribution, where **D** is a known block diagonal matrix representing the covariance between permanent environmental components. The **D** matrix was calculated by this formula: **D** = **E** ⊗ **I**, where **E** is a 3 × 3 matrix representing the covariance between the set of 3 time points for each individual.

### Identification and annotation of candidate genes

The UMD3.1 genome assembly was used to located genes for annotation, and the QTLdb database (http://www.animalgenome.org) was applied to search for related QTL regions.

## Supplementary information

**Additional file 1: Figure S1.** The strengths of genome-wide association studies (GWAS) are illustrated by the Manhattan plots on the left panel. The deviations of the signals from null hypothesis are illustrated as the Quantile-Quantile (QQ) plots on the right panel. The negative logarithms of the observed (y axis) and the expected (x axis) *P* values are plotted for each SNP (dot). GWAS were performed six body size traits months 6, 12 and 18 after birth separately. Each analysis is labeled as trait (BH or HH) and month on the far right. The number neighboring each trait indicates the age of measurement (e.g., BH6 = Body Height at 6 months). The 29 chromosomes are color coded.

**Additional file 2: Figure S2.** The strengths of genome-wide association studies (GWAS) are illustrated by the Manhattan plots on the left panel. The deviations of the signals from null hypothesis are illustrated as the Quantile-Quantile (QQ) plots on the right panel. The negative logarithms of the observed (y axis) and the expected (x axis) P values are plotted for each SNP (dot). GWAS were performed six body size traits months 6, 12 and 18 after birth separately. Each analysis is labeled as trait (BH or HH) and month on the far right. The number neighboring each trait indicates the age of measurement (e.g., BH6 = Body Height at 6 months). The 29 chromosomes are color coded.

**Additional file 3: Table S1.** Descriptive statistics of 133 phenotypic records in LONG-GWAS method.

## Data Availability

We confirm that all raw data underlying our findings are publicly available without restriction. Data is available from the Dryad Digital Repository: doi:10.5061/dryad.4qc06.

## Abbreviations

GWAS: Genome-wide association study
BH: Body height
BL: Body length
HH: Hip height
HS: Heart size
AS: Abdominal size
CS: Cannon bone size
SNP: Single nucleotide polymorphism
QTL: Quantitative trait loci;
PCA: Principal components analysis
CMLM: Compressed mixed linear model;
GAPIT: Genome Association and Prediction Integrated Tool
BTA: *Bos Taurus* chromosome
LONG-GWAS: Longitudinal GWAS
