# Supplementary figures and images for: Multiple association analysis of loci and candidate genes that regulate body size at three growth stages in Simmental beef cattle
Source: BMC Genet. 2020 Mar 14;21:32. doi: 10.1186/s12863-020-0837-6 (PMC7071762; doi:10.1186/s12863-020-0837-6)

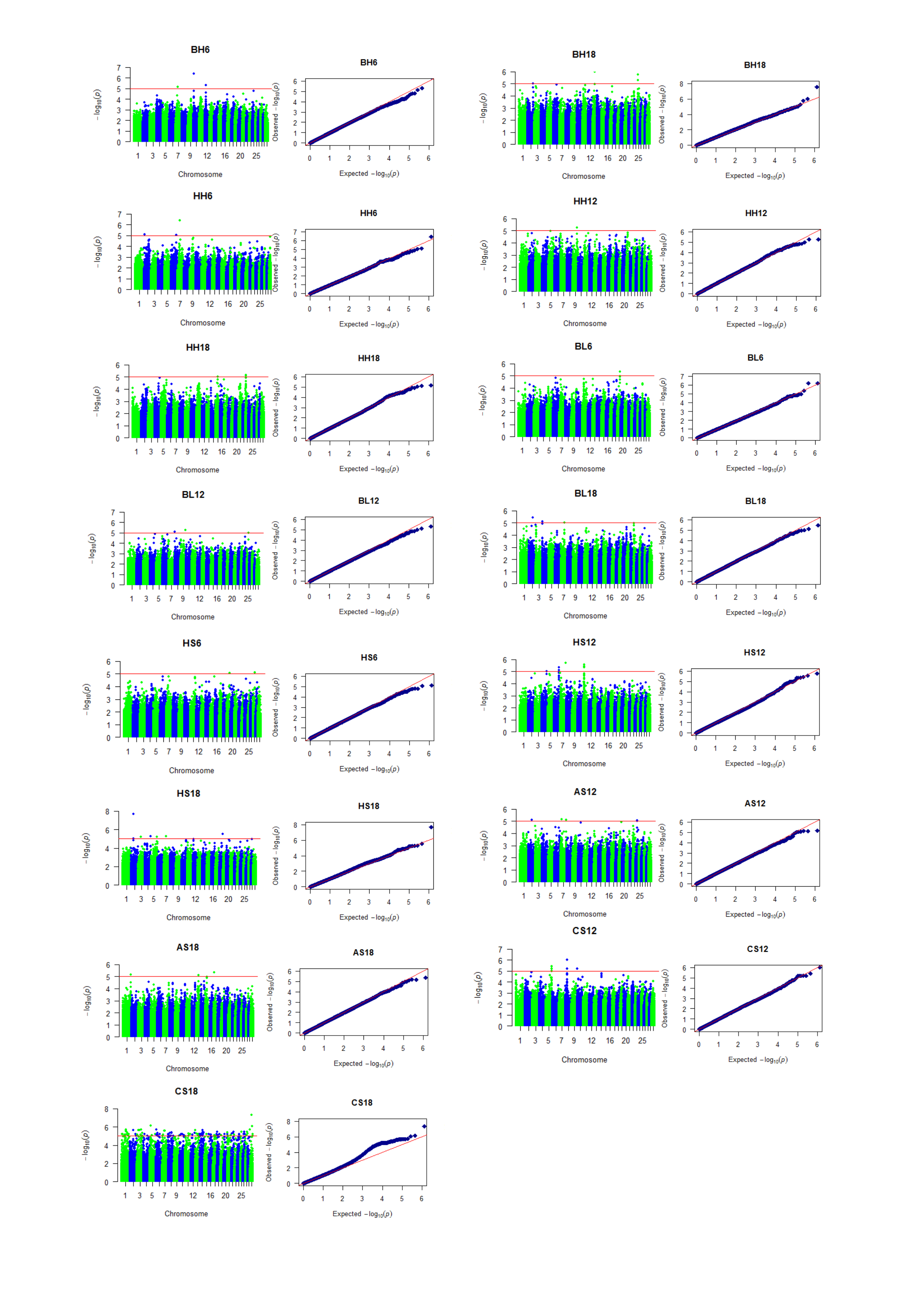

Supplement: Supplementary file 1 — Additional file 1: Figure S1. The strengths of genome-wide association studies (GWAS) are illustrated by the Manhattan plots on the left panel. The deviations of the signals from null hypothesis are illustrated as the Quantile-Quantile (QQ) plots on the right panel. The negative logarithms of the observed (y axis) and the expected (x axis) P values are plotted for each SNP (dot). GWAS were performed six body size traits months 6, 12 and 18 after birth separately. Each analysis is labeled as trait (BH or HH) and month on the far right. The number neighboring each trait indicates the age of measurement (e.g., BH6 = Body Height at 6 months). The 29 chromosomes are color coded. [file 12863_2020_837_MOESM1_ESM.tiff]

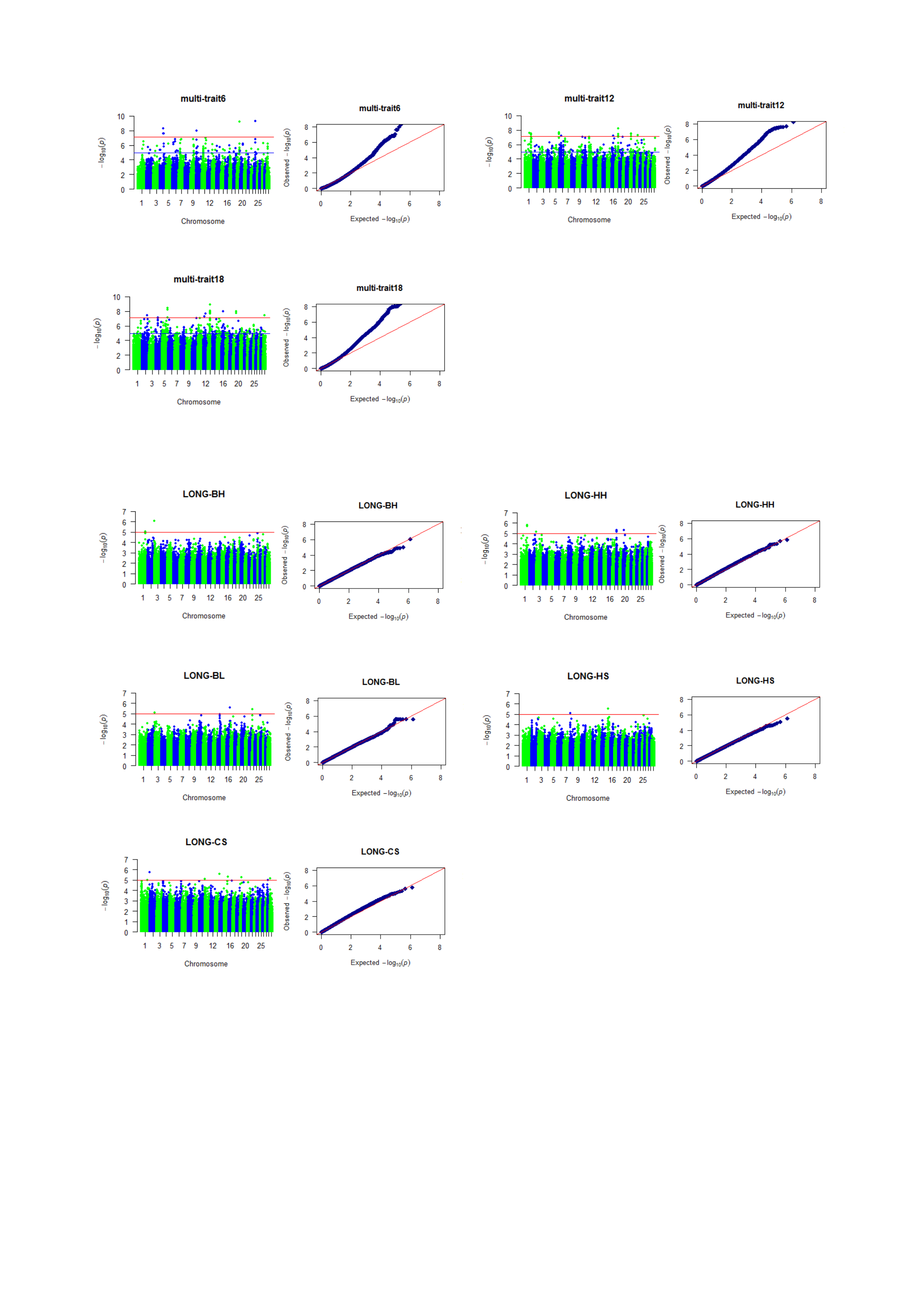

Supplement: Supplementary file 2 — Additional file 2: Figure S2. The strengths of genome-wide association studies (GWAS) are illustrated by the Manhattan plots on the left panel. The deviations of the signals from null hypothesis are illustrated as the Quantile-Quantile (QQ) plots on the right panel. The negative logarithms of the observed (y axis) and the expected (x axis) P values are plotted for each SNP (dot). GWAS were performed six body size traits months 6, 12 and 18 after birth separately. Each analysis is labeled as trait (BH or HH) and month on the far right. The number neighboring each trait indicates the age of measurement (e.g., BH6 = Body Height at 6 months). The 29 chromosomes are color coded. [file 12863_2020_837_MOESM2_ESM.tiff]
