# Supplementary material for: Multiple association analysis of loci and candidate genes that regulate body size at three growth stages in Simmental beef cattle
Source: BMC Genet. 2020 Mar 14;21:32. doi: 10.1186/s12863-020-0837-6 (PMC7071762; doi:10.1186/s12863-020-0837-6)
Supplement: Supplementary file 3 — Additional file 3: Table S1. Descriptive statistics of 133 phenotypic records in LONG-GWAS method. [file 12863_2020_837_MOESM3_ESM.docx]

Table S1 Descriptive statistics of 133 phenotypic records in LONG-GWAS method.

| Month | Trait (cm) | Mean | Min. | Max. | SD |
| --- | --- | --- | --- | --- | --- |
| 6 | BH | 101.01 | 82 | 120 | 9.145 |
|  | HH | 109.77 | 88 | 128 | 9.029 |
|  | BL | 104.96 | 78 | 134 | 9.707 |
|  | HS | 126.79 | 96 | 162 | 13.92 |
|  | AS | 141.42 | 97 | 188 | 15.93 |
|  | CS | 16.17 | 12 | 20 | 1.748 |
| 12 | BH | 115.92 | 99 | 133 | 6.914 |
|  | HH | 122.45 | 105 | 138 | 6.930 |
|  | BL | 128.54 | 104 | 150 | 8.876 |
|  | HS | 167.02 | 137 | 202 | 12.59 |
|  | AS | 195.99 | 163 | 237 | 14.50 |
|  | CS | 18.05 | 16 | 22 | 1.235 |
| 18 | BH | 125.98 | 116 | 143 | 4.291 |
|  | HH | 133.29 | 116 | 143 | 3.023 |
|  | BL | 144.20 | 115 | 170 | 7.957 |
|  | HS | 187.60 | 169 | 205 | 7.306 |
|  | AS | 218.13 | 193 | 242 | 8.692 |
|  | CS | 20.54 | 17 | 23 | 1.086 |

SE standard error, BH body height, BL body length, HH hip height, HS heart size, AS abdominal size, CS cannon bone size

^a^Number of animal with phenotypes
